# Evaluation of mushroom-shaped allograft for unstable proximal humerus fractures

**DOI:** 10.1007/s00402-020-03715-w

**Published:** 2020-12-23

**Authors:** Lukas Dankl, Werner Schmoelz, Romed Hoermann, Simon Euler

**Affiliations:** 1 Department of Orthopaedics and Traumatology, Medical University of Innsbruck, Anichstraße 35, 6020 Innsbruck, Austria; 2 Division Clinical and Functional Anatomy, Medical University of Innsbruck, Innsbruck, Austria; 3 Trauma and Orthopedic Surgery, Sanatorium Kettenbruecke der Barmherzigen Schwestern GmbH, Innsbruck, Austria

**Keywords:** Proximal humerus fracture, Varus displacement, Allograft, Bone grafting, Angular stable locking plate

## Abstract

**Introduction:**

Proximal humerus fractures are common injuries of the elderly. Different treatment options, depending on fracture complexity and stability, have been recommended in the literature. Particularly for varus displaced fractures with a lack of medial support, and patients suffering from osteoporosis, structural allografts can be used to enhance the stability of the construct. An individually shaped allograft has been suggested in the literature and investigated in a clinical setting. However, biomechanical properties have yet to be evaluated.

**Materials and methods:**

Twenty-four fresh-frozen humeri and 12 femoral heads were obtained, and an unstable three-part fracture of the humeral head was simulated. Fracture fixation was achieved by using a locking plate in both groups. In the test group, a mushroom-shaped allograft was tailored out of a femoral head to individually fit the void inside the humeral head. Specimens were fitted with a 3D motion analysis system and cyclically loaded with a stepwise increasing load magnitude in a varus-valgus bending test until failure or up to a maximum of 10,000 load cycles.

**Results:**

The mushroom group reached a significantly higher number of load cycles (8342; SD 1,902; CI 7133–9550) compared to the control group (3475; SD 1488; CI 2530–4420; *p* < 0.001). Additionally, the test group showed significantly higher stiffness values concerning all observational points (*p* < 0.001).

**Conclusion:**

This mushroom-shaped allograft in combination with a locking plate significantly increased load to failure as well as stiffness of the construct when exposed to varus-valgus bending forces. Therefore, it might be a viable option for surgical treatment of unstable and varus displaced proximal humerus fractures to superiorly prevent loss of reduction and varus collapse.

## Introduction

Proximal humerus fractures are common among the elderly suffering low energy trauma [1, 2]. They represent the second most common fractures of the upper limb in patients older than 65 years [3] and predominantly affect women [1, 4]. Minimally displaced and stable fractures can be treated conservatively [5, 6]. For displaced and unstable fractures, however, surgical treatment ranging from closed reduction and k-wire fixation, locking nail or locking plate fixation to shoulder arthroplasty has been recommended [5–7]. While locking plates show good clinical results [5, 8], osteoporotic bone stock and a low local bone mineral density (BMD) in the humeral head have been described as predictive factors for failure of the fracture fixation [9, 10]. Varus collapse of the head fragment with screw cut-out is the most common reason for revision surgery [11, 12]. Additionally, a varus deformity of more than 45° significantly decreases the supraspinatus tendon efficiency and increases arm elevation forces [13]. Stable fixation of the medial hinge seems to be a key factor to avoid varus collapse, and neglecting this structure may potentially lead to failure of the surgically fixed construct [2, 6, 14–17].

Different techniques to enhance the stability of fracture fixation in poor bone stock have been described in the literature. In a biomechanical study, Unger et al. showed a significant increase in load cycles until failure for varus bending as well as axial torque for in situ augmentation of cannulated screws in proximal humeral fractures [18]. In a clinical study, Knierzinger et al. observed a low secondary displacement rate but a high rate of avascular necrosis using this technique [19]. Gardner et al. proposed an intramedullary fibula strut graft in combination with a locking plate that showed promising clinical results in a case series of seven patients. They observed progressive incorporation of the graft after three to four months [20]. More recently, Kim et al. evaluated this graft in a retrospective series of 63 patients and observed improved clinical outcomes [21]. The biomechanical advantages of a fibula graft in unstable proximal humerus fractures have been demonstrated by different authors with increased load to failure and less interfragmentary displacement [22–26]. Kim et al. suggested the use of autologous iliac bone graft to restore the medial cortical support and likewise showed good clinical results in a case series of 21 patients.[27]. All these techniques, however, do not fully utilize the entire possible area of contact between the graft and the humeral head [28]. Therefore, Euler et al. suggested an individually tailored mushroom-shaped graft to exactly fit into the bony defect of the humeral head. They were able to show promising clinical results, even in a high-risk patient group. Bone union was observed after a mean follow-up of 28.5 months in all cases with satisfactory functional outcome [28].To our knowledge, this mushroom-shaped graft has not yet been tested biomechanically.

Therefore, the aim of this study was to biomechanically evaluate this individually shaped graft. We hypothesized that compared to the control group, a supportive mushroom-shaped bone allograft in combination with locking plate fixation of unstable proximal humerus fractures results in a significantly higher stiffness and number of load cycles to failure.

## Materials and methods

### Specimens

Twenty-four paired fresh-frozen humeri of 12 donors (6 male and 6 females, mean age 71.6, years, range 51 to 91) and 12 femoral heads (mean age 69.2 years (range 51 to 78)) were obtained from the local anatomical department. A qCT-scan (GE Lightspeed VCT 16, Milwaukee, USA) calibrated with the European Forearm Phantom (EFP) (EFP‐Phantom, QRM GmbH, Möhrendorf, Germany) was performed to rule out any prior surgery as well as preexisting pathologies. The BMD was calculated according to the technique described by Krappinger et al. [29]. All specimens were vacuum sealed in plastic bags and stored at  − 20° C. Before testing, specimens were thawed at 6 °C for 12 h and all soft tissue was removed.

The humeri of each pair were randomly assigned into one of two groups to allow a paired comparison of the two techniques in similar bone morphology: The “Mushroom Group” (MG) was treated with a locking plate fixation in combination with a bony allograft, while the “Control Group” (CG) was treated with a locking plate alone.

### Fracture creation and surgical technique

**Fig. 1:**
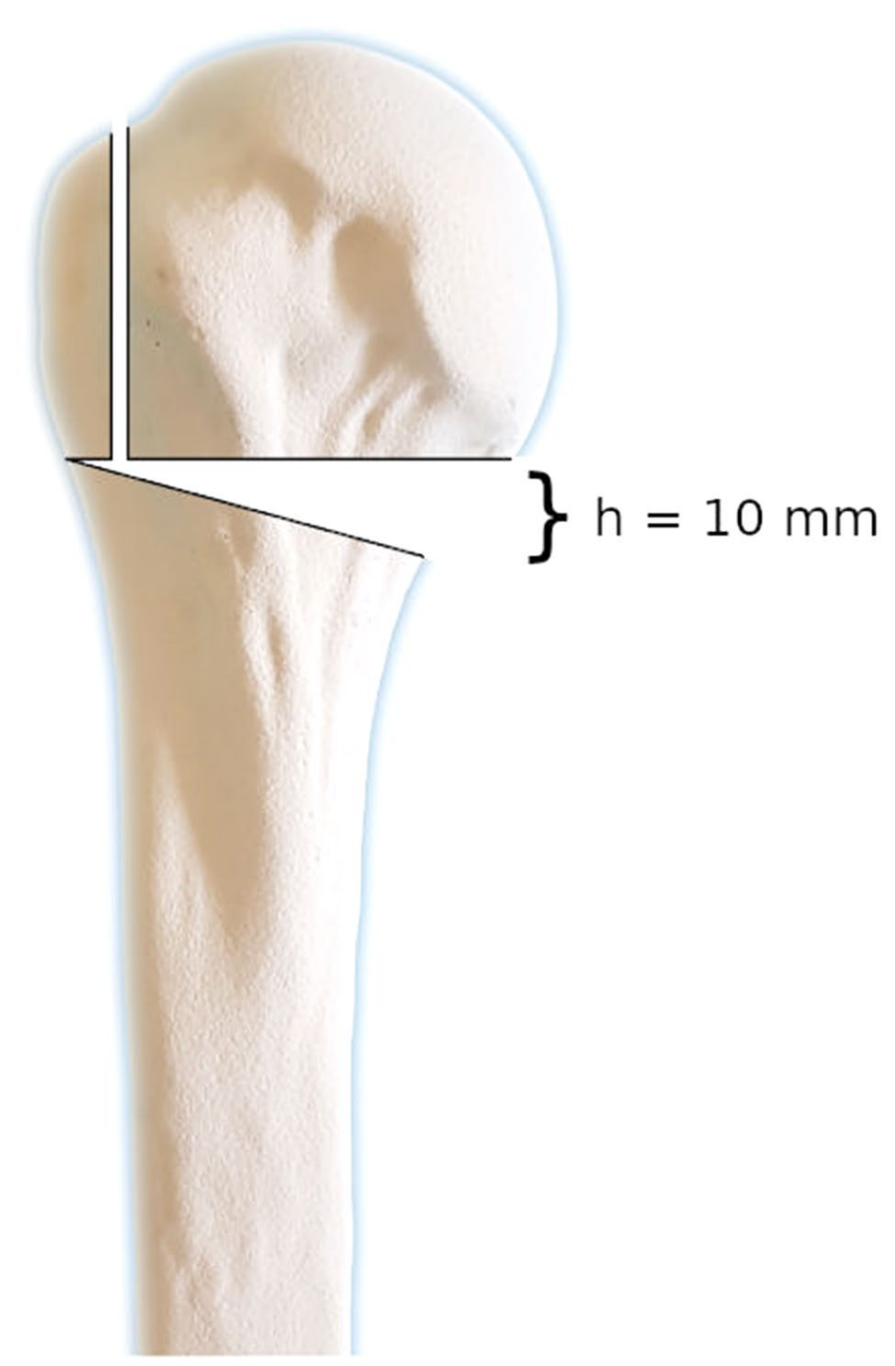
A three-part fracture was created performing an osteotomy at the greater tuberosity, and a V-shaped osteotomy of the surgical neck. The height of the medial osteotomy gap was 10 mm

An unstable three-part fracture of the humeral head was simulated by an osteotomy of the greater tuberosity laterally to the sulcus intertubercularis and a V-shaped osteotomy at the anatomical neck. Prior to the osteotomy, the fracture lines were marked on the specimen. The first osteotomy was positioned at the level of the cartilage-bone border rectangularly to the shaft axis. The second osteotomy was angulated to create a wedge-shaped fracture gap with a height of 10 mm on the medial side. (Fig. 1). The osteotomy was performed using an oscillating saw.

**Fig. 2:**
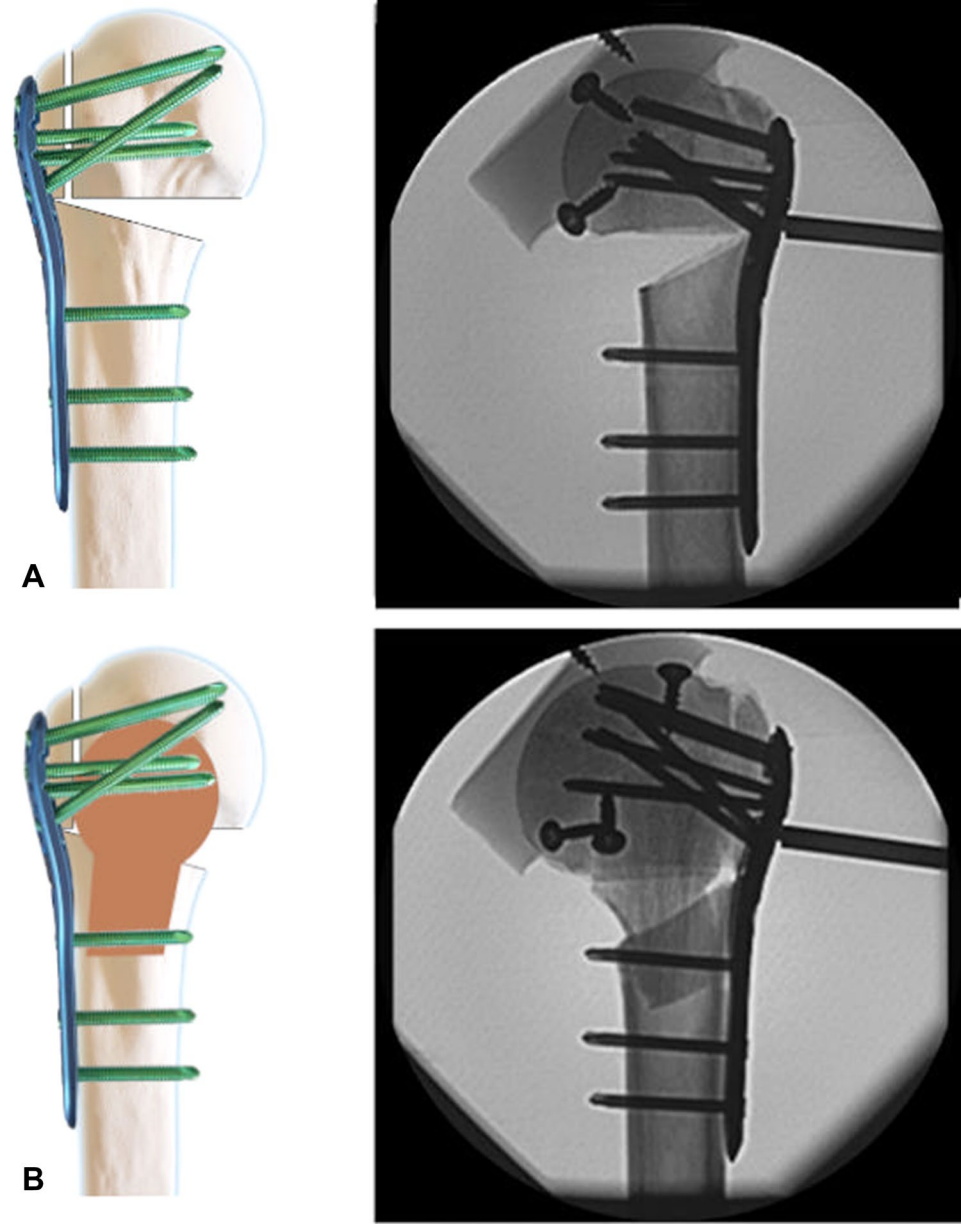
Schematic and X-ray illustration of the two test groups. **a**: In the control group (CG), the fracture was fixed with a locking plate alone. **b**: In the mushroom group (MG), a mushroom-shaped allograft was positioned inside the humeral head and the shaft. The B-section screws penetrated the graft

Graft implantation was performed by the same surgeon in all specimens. Fracture fixation was achieved using a locking plate (PHILOS, Synthes GmbH, Oberdorf, Switzerland) with Ø 3.5-mm self-tapping angular stable locking screws (Synthes GmbH, Oberdorf, Switzerland). All plates were implanted according to the manufacturer’s guidelines. In both groups, the locking plate was attached to the humeral shaft with three screws in the F, G and H section holes. The head fragment was fixed with two screws in the A, B and C section holes, respectively. The length of the screws was determined using a measuring tool provided by the manufacturer. The screws were positioned with the tip of the screw 5–8 mm below the joint surface (Fig. 2).

In the MG, a mushroom-shaped allograft was tailored freehand out of a femoral head. For graft preparation, all cartilage and cortical bone was removed from the femoral head using an oscillating saw. The graft was then carefully shaped with a rongeur and the oscillating saw to fit the void inside the humeral head as well as to anchor in the humeral shaft as described by Euler et al. [28]. When anatomical reduction was achieved, the locking plate was fixed as described previously (Fig. 2).

Following implantation and fixation, all specimens were cut to a humeral length of 150 mm and embedded in polymethylmethacrylate (PMMA) cement (Technovit 3040, Heraeus Kulzer GmbH, Wehrheim, Germany) for fixation in the material testing machine.

Prior to and after testing, anterior–posterior and medial–lateral radiographs were obtained.

### Biomechanical test setup

**Fig. 3:**
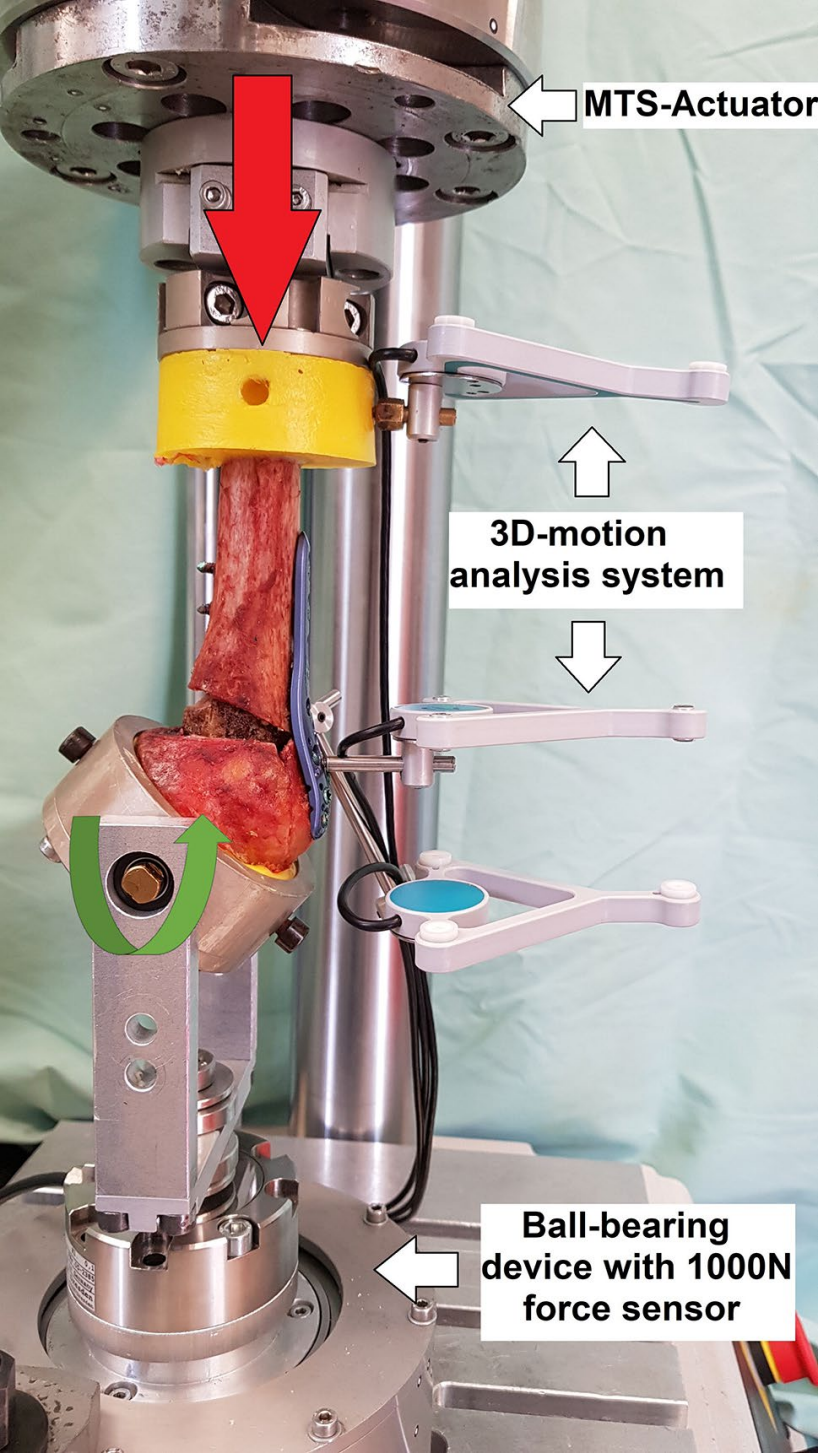
Test setup for varus-valgus bending. The red arrow indicates the load application. The green arrow indicates the axis of rotation for the humeral head. The construct was mounted on a ball-bearing device to minimize shear forces

Biomechanical testing was carried out using a biaxial servo‐hydraulic material testing machine (MTS, 858 MiniBionix II, MN, USA). Relative fracture gap motion between the locking plate, the humeral head and humeral shaft was recorded by an ultrasound-based 3D motion analysis system (Zebris Medical GmbH**,** Isny, Germany). Three sensors were attached to each specimen. This setup was allowed for measurement of humeral head tilt relative to the locking plate between the proximal and the middle sensor as well as bending of the locking plate between the middle and the distal sensor (Fig. 3).

For testing, the embedded distal humeral shaft was fixed to the actuator of the servo-hydraulic testing machine. The humeral head was fixed to a special rig on a ball‐bearing device to allow rotation in varus and valgus direction and to minimize shear forces (Fig. 3).

Specimens were cyclically loaded in a varus-valgus bending test until failure or up to a maximum of 10,000 load cycles. Initially, the loading ranged from  − 10 N (valgus) to 50 N (varus), while the load magnitude in varus was increased stepwise by 5 N every 100 load cycles up to 550 N. The stepwise increase in load was applied displacement controlled (5 mm/s) with force limits. This resulted in a varus load magnitude of 55 N after 100 load cycles, 100 N after 1,000 load cycles and 150 N after 2,000 load cycles etc.

Angulation of the humeral head relative to the locking plate and bending of the locking plate under varus and valgus stress was measured by an ultrasound-based 3D motion analysis system, and the maximum angulation in the first load cycle at the beginning of each load step (every 100 cycles) was determined. Additionally, the axial displacement of the actuator was recorded by the software of the material testing machine.

Failure was defined as an increase in head fragment varus angulation of more than 0.5 degrees within 100 load cycles, head fragment varus tilt of more than eight degrees relative to the starting position, bending of the locking plate of more than eight degrees relative to the starting position or axial displacement of the actuator of more than 10 mm (and therefore a complete occlusion and collapse of the osteotomy gap) [18].

Stiffness was calculated at the beginning of the test protocol (50 N), at 100 N, at 125 N and at the last load step before failure. To calculate stiffness, maximum load range from valgus to varus within one cycle and the corresponding axial displacement were used.

Figures were created using GIMP (GNU Image Manipulation Program, Version 2.8.10, CC BY-SA 4.0). Statistical analysis was performed using SPSS (IBM SPSS Statistics, Version 24.0.0.1, IBM Corporation, Armonk, New York, USA). Mean values, standard deviations (SD) and 95% confidence intervals (CI) were calculated. A one-sample Kolmogorov–Smirnov test was used to screen for normal distribution. A paired Student’s t test was used for the comparison of the two groups. The significance level was set to p < 0.05.

## Results

The random assignment resulted in five right humeri and seven left humeri in the MG, and seven right humeri and five left humeri in the CG. The BMD of the two groups did not differ significantly (*p* = 0.899, MG 114.6 mg/cm^3^ (SD 28.0; CI 96.8–132.4), CG 114.9 mg/cm^3^ (SD 31.8; CI 94.7–135.1)). The mean BMD of the femoral heads was 263.5 mg/cm^3^ (SD 42.1; CI 236.8–290.3).

**Fig. 4:**
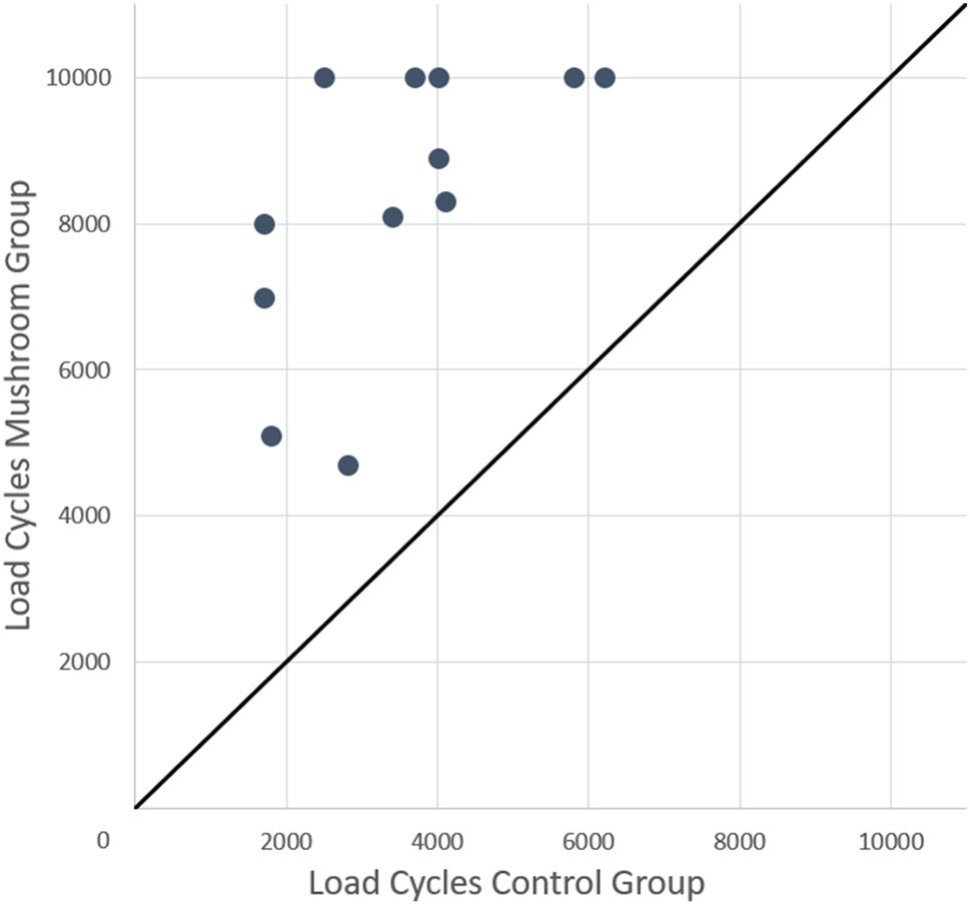
Scatter plot of the number of load cycles to failure. One dot marks two humeri of one pair with the control group (CG) on the x-axis and the mushroom group (MG) on the y-axis

**Fig. 5:**
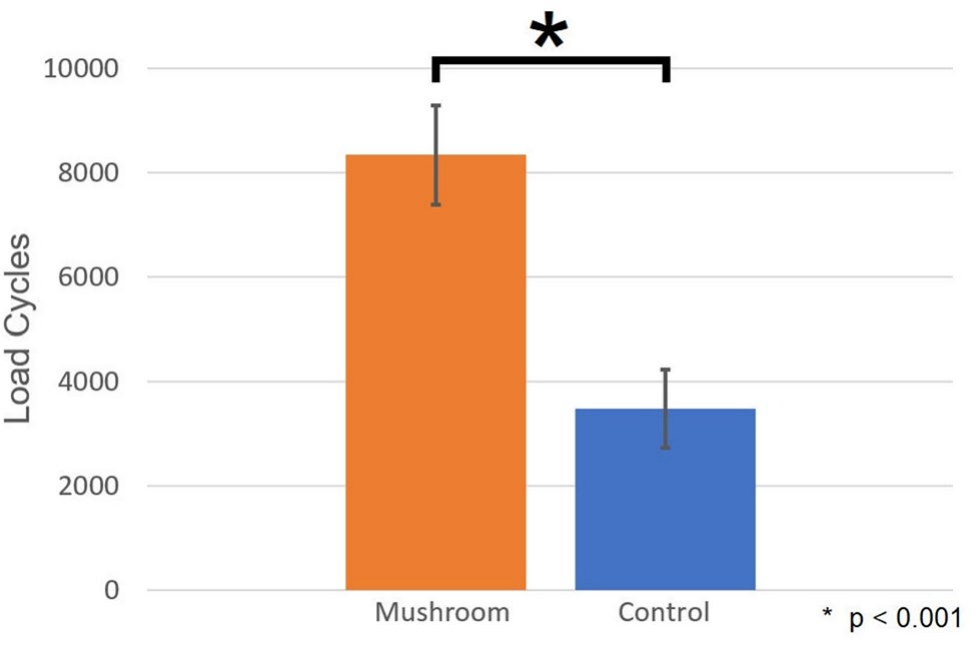
Bar plot of the number of load cycles to failure. Mean and standard deviation (error bars) of number of load cycles until failure for MG and CG. The asterisk above the bars indicates a significant difference (*p* < 0.05)

In the MG, the load magnitude and number of load cycles until failure were higher in each individual pair (Fig. 4). In the varus bending test, specimens of the MG reached a mean of 8342 load cycles (SD 1902; CI 7133–9550) until failure, while specimens in the CG reached a mean of 3475 load cycles (SD 1488; CI 2530–4420) until failure. This corresponds to a load of 467.1 N (SD 95.1; CI 406.6–527.5) and 223.8 N (SD 74.4; CI 176.5–271.0), respectively. The difference was significant (*p* < 0.001) (Fig. 5). In the MG, seven specimens failed because of more than 0.5 degrees varus head angulation within 100 load cycles. Five specimens completed the cyclic load protocol without reaching any of the failure criteria. In the CG, nine specimens failed due to more than 0.5 degrees varus head angulation within 100 load cycles, one specimen showed more than eight degrees of varus angulation relative to the starting position and two specimens showed more than eight degrees of plate bending.

The MG showed significantly higher stiffness values compared to the CG at each defined observation point. On average, the mean stiffness was found to be 4.5 times higher (*p* < 0.001) when the mushroom allograft was applied (Table 1).

**Table 1:** Mean, SD and CI of the initial stiffness, the stiffness at 100 N, at 125 N and at the last load step before failure for the mushroom group and the control group are shown in N/mm

	Initial stiffness in N/mm	Stiffness in N/mm at 100 N	Stiffness in N/mm at 125 N	Stiffness in N/mm at last load step before failure
Mushroom group (MG)	**425.5**	**448.2**	**470.3**	**268**
	SD 172.1	SD 210.6	SD 211.2	SD 138.4
	CI 316.1–534.9	CI 314.4–582	CI 336.1–604.5	CI 180–356
Control group (CG)	**99.2**	**96.1**	**91.3**	**71.1**
	SD 31.8	SD 36.3	SD 34.9	SD 32.4
	CI 79–119.4	CI 73–119.1	CI 69.1–113.4	CI 50.5–91.7
*p*-value	< 0.001	< 0.001	< 0.001	< 0.001

## Discussion

The most important finding of this study is that the construct’s load to failure was more than double by the use of a mushroom-shaped allograft. The allogenic bone graft increased the load to failure in every single testing pair. When comparing stiffness, the presence of an allograft increased the observed mean stiffness values up to five times compared to the conventional treatment. At the last load step before failure, the stiffness was nearly four times higher than in the CG. The most common type of failure was loosening of the screws inside the humeral head and therefore a varus collapse of the construct. This resembles the failure mode observed in a clinical setting [11, 12].

Mathison et al. [22] biomechanically investigated the effect of an intramedullary fibula graft on a two-part humeral head fracture model. They showed a 1.72-fold increase in load to failure and an average 3.84-fold increase in stiffness when a graft was used for augmentation. These values are slightly lower compared to our findings. This might be due to the shape of the graft. While the fibula graft has a small contact area resulting in stress concentrations at the head–graft interface, the mushroom-shaped graft offers a larger contact area and can distribute the load more evenly, avoiding stress concentrations. The most common failure modes of the present study were screw cut-out with subsequent varus tilting of the humeral head, or plate bending. Mathison et al. reported humeral head split fractures and humeral shaft fractures as main failure modes. These differences are most likely due to variations in the test setup and the loading jig. While Mathison et al. applied the load to the humeral shaft and fully constrained the humeral head, in the present study, the load was applied to the humeral head and the humeral shaft was only partially constrained [22].

Osterhoff et al. [24] showed a decreased intercycle fracture gap motion and decreased fragment migration when cyclically loading two-part humeral fractures reinforced with a fibula graft in a custom shoulder testing device that allowed varus collapse. They used synthetic bones and reported only results of the nondestructive cyclic loading. They also showed a higher stability for their fibular graft; however, compared to the mushroom graft group of the present study, the axial displacement was higher.

Bae et al. [26] showed a mean maximum failure load of 1985.54 N when loading fresh-frozen human humeri with a two-part fracture and a fibular strut graft. The contralateral side, treated with a locking plate alone, reached a mean of 1291.83 N until failure. These values are much higher than in our study and in other studies described in the literature [18, 22]. This may be due to the design of the test setup. The authors orientated the humeri vertically and applied a vertical load directly onto the humeral head parallel to the long axis of the humeral shaft [26]. In our test setup, we used a ball-bearing device to minimize shear forces and a special rig to allow for varus collapse, as this is a common mode of fixation failure [11, 12] (Fig. 3). This setup more closely resembles physiological loading of the humerus by simulating a varus moment comparable to in vivo forces created by the rotator cuff [30]. In addition, it allows for screw loosening and fixation failure similar to the observations in clinical reality.

When comparing our results to studies investigating the effect of in situ augmentation, Unger et al. [18] showed very similar load to failure values for the non-augmented control group in a comparable varus compression test setup. When augmentation was performed, the authors observed a 1.4-fold increase in load to failure magnitude with varus compression [18], while we observed a 2.1-fold increase when the mushroom graft was applied.

We observed a significant increase in stiffness and a significant decrease in plate bending when the mushroom allograft was applied. This supports our theory that some part of the applied force gets absorbed by the graft. The graft acts as a counter bearing, giving the screws healthy bone structure to purchase. This may contribute positively to the construct’s overall rigidity.

Euler et al. [28] applied this kind of mushroom-shaped allograft in a two-part fracture setting on a high-risk patient population with large bone defects as a single stage surgery. After a median follow-up of 28.5 months, the authors observed a median Constant–Murley Score of 72.0 points and a persistent average decrease in varus angulation of 38° compared to the preoperative status in a case series of ten patients. All fractures healed without relevant loss of reduction. The mean loss of flexion and abduction was acceptable [28]. We were now able to demonstrate the biomechanical advantages of this mushroom-shaped graft in an even more unstable three-part fracture setting. Some authors recommend primary hemiarthroplasty or reverse shoulder arthroplasty in unstable, complex and displaced fractures, especially in older individuals [6]. The reconstruction of the medial hinge seems indispensable to avoid varus collapse and therefore failure of the fixation [2, 6, 14–17]. This graft could be a surgical alternative for patients with complex and displaced fractures in order to avoid primary arthroplasty.

Like in all biomechanical studies using fresh-frozen specimens, this study has inherent limitations. The specimens were cleaned of all soft tissue and loaded by a material testing machine. Although our test setup aimed to simulate physiological loading conditions, only an axial force superimposed by a varus moment was applied. True physiological muscle pull of the shoulder girdle and the influence of soft tissue on the initial stability were not implemented in this biomechanical setting. Secondly, we did not use calcar screws (E-section screws) in our test setup. Ponce et al. demonstrated an increase in mean load to failure by 31% in proximal humerus fractures without medial comminution when calcar screws were applied [17], while Shin et al. could not replicate this finding in a two-part fracture model [31]. In a clinical setting, however, the application of calcar screws may not always be possible. We also hypothesized that implementing calcar screws in our test setup would only result in a higher mean load to failure in both groups. Therefore, we decided against the use of E-section screws to demonstrate the supporting effect of a mushroom-shaped allograft without this additional stabilizing influence. Lastly, our findings only reflect the fracture fixation initially after surgery. The effect of in vivo bone healing and remodeling may alter the biomechanical findings over time.

## Conclusion

In conclusion, this biomechanical study shows that a mushroom-shaped allograft in combination with a locking plate fixation significantly increases the load to failure as well as the stiffness of the construct when compared to conventional locking plate fixation in patients with low BMD, as well as osteoporotic bone stock. It may be a viable alternative to prevent varus collapse when addressing unstable proximal humerus fractures with large bone defects or lack of medial cortical stability.
